# Profitability, energetics and GHGs emission estimation from rice-based cropping systems in the coastal saline zone of West Bengal, India

**DOI:** 10.1371/journal.pone.0233303

**Published:** 2020-05-21

**Authors:** Krishnendu Ray, Piyali Sen, Rupak Goswami, Sukamal Sarkar, Koushik Brahmachari, Argha Ghosh, Manoj Kumar Nanda, Mohammed Mainuddin

**Affiliations:** 1 Sasya Shyamala Krishi Vigyan Kendra, Ramakrishna Mission Vivekananda Educational and Research Institute, Kolkata, West Bengal, India; 2 Integrated Rural Development and Management Faculty Centre, Ramakrishna Mission Vivekananda Educational and Research Institute, Kolkata, West Bengal, India; 3 Department of Agronomy, Bidhan Chandra Krishi Viswavidyalaya, West Bengal, India; 4 Department of Agricultural Meteorology and Physics, Bidhan Chandra Krishi Viswavidyalaya, West Bengal, India; 5 Black Mountain LaboratoriesCanberra ACT, CSIRO Land and Water, Canberra, Australia; Harran University, TURKEY

## Abstract

This study compares thirteen rice-based cropping systems in the coastal part of West Bengal, India in terms of productivity, profitability, energetics, and emissions. Information on the crop management practices of these systems was collected on 60 farms through a questionnaire survey. Rice-bitter gourd system was observed to have the highest system yield (49.88 ± 4.34 tha^−1^yr^−1^) followed by rice-potato-ridge gourd (37.78 ± 2.77 tha^−1^yr^−1^) and rice-potato-pumpkin (36.84 ± 2.04 tha^−1^yr^−1^) systems. The rice-bitter gourd system also recorded the highest benefit:cost ratio (3.92 ± 0.061). The lowest system yield and economics were recorded in the rice-fallow-fallow system. Rice-sunflower system recorded highest specific energy (2.54 ± 0.102 MJkg^−1^), followed by rice-rice (2.14 ± 0.174 MJkg^−1^) and rice-fallow-fallow (1.91 ± 0.327 MJkg^−1^) systems, lowest being observed in the rice-bitter gourd (0.52 ± 0.290 MJkg^−1^) and rice-pointed gourd (0.52 ± 0.373 MJkg^−1^) systems. Yield-scaled GHGs (YSGHG) emission was highest (1.265 ± 0.29 t CO_2eq_t^−1^ system yield) for rice-fallow-fallow system and was lowest for rice-vegetable systems. To estimate the uncertainty of the YSGHG across different systems under study, Monte-Carlo Simulation was performed. It was observed that there was a 5% probability of recording YSGHG emission > 1.15 t CO_2eq_t^−1^ system yield from different cropping systems in the present experiment. Multiple system properties such as productivity, economics, energy, and emission from all rice-based systems taken together, the rice-vegetable system performed consistently well across parameters and may be practised for higher economic returns with judicious and sustainable utilization of resources in the coastal saline tracts of the region.

## Introduction

Sustainable and climate-resilient forms of agriculture have become an imperative for maintaining a balance between increasing production and limited resources available for agricultural systems [1] and this concern is extremely crucial for rice (*Oryza sativa* L.)-based cropping systems tended by 300 million people in 140 million ha lands in Asia [2]. It is argued that synergistic coproduction of agricultural and natural capital is possible [3] and such synergy can be achieved by enhanced resource-use efficiency, the substitution of inputs, and the design of sustainable farming systems [3,4]. However, even before we incorporate the existing scientific knowledge into the development and scaling up of sustainable rice-based cropping practices, it would be pragmatic to take account of the existing cropping systems in terms of multifaceted parameters. Judging cropping systems has long been predominated by productivity and profitability parameters until recently when more holistic and overarching parameters (such as energy and emission) are addressed empirically [5] with an explicit focus on ecosystem services [6]. Assessment of rice-based cropping systems in terms of multiple criteria such as economics, energy efficiency, and global warming potential may be a starting point of finding and developing sustainable cropping and farming systems for a region.

From the perspective of system analysis, that focuses on the design of complex systems, three contexts are commonly addressed *viz*. input analysis (when output and device are given), output analysis (when input and device are given), and system design (when input and outputs are given) [7]. The intervention for sustainable agricultural systems may be–(a) input choice and management, or (b) diagnosis of the performance of agricultural systems, or (c) design of farming systems that achieve certain levels of systems performance by using certain levels of inputs [8]. Our study falls under the second category that aims to diagnose/describe the rice-based cropping systems in terms of economics, energetics, and emission that can either be promoted among farmers or be taken up for input analysis and system design projects. Unlike many cropping system analysis frameworks used as a part of cropping system models, we limit our study to the assessment of existing popular rice-based cropping systems and judge their relative strengths and weaknesses in terms of energetics, global warming potential, and economics. Moreover, energy use, emission of GHGs, and economics of a cropping system are interrelated [9] and they are best understood when studied together.

Natural and anthropogenic emission of greenhouse gases (GHGs) such as nitrous oxide (N_2_O), methane (CH_4_), and carbon dioxide (CO_2_) has been recognized as the key reason behind Global climate change [10, 11]. Agriculture has now become a serious contributor (by contributing about 20%) to such anthropogenic GHGs emission [11], and it shares about 60% and 50% of total N_2_O and CH_4_, two most potent anthropogenic GHGs [12]. In India, agriculture shares about 23% of the total emission of GHGs from all possible sources and this accounts for about 12% and 67.1% of emission from World and South-Asian agriculture, respectively [13]. Rice-based double or triple cropping systems have always been predominant in India [14] which includes growing of cereal, pulse, oilseed, vegetable, etc. along with post-rainy season rice crop. Because of its extensive practice, rice-based systems have repeatedly been analysed and found to be a contributor to major anthropogenic sources of CH_4_ and N_2_O [5, 15]. According to [16], rice fields can alone contribute to about 19% and 11% of total N_2_O and CH_4_ emissions, respectively. Thus, it is imperative to find out suitable rice-based systems for different agro-ecological regions of the country where the emission of GHGs will be comparatively lower, without compromising the system yield and economics.

Crop productivity results transformation of solar energy into metabolizable energythrough photosynthetic pathways [17]. Thus, system productivity and profitability mainly depend on farming practices, which include the use of seed, fertilizer, pesticide, irrigation and labour energy sustainablly. Rice-based cropping systems include several management practices such as land preparation, fertilizer and pesticide application, irrigation, harvesting and transportation. Thus, proper utilization of available resources necessitates analyzing energy input and output of different rice-based systems [5, 18].

The coastal saline tract of the state West Bengal is considered as one of the most resource-constrained areas of the country. It experiences both soil salinity and inundation problems with elevation within polder. Besides, the availability of freshwater from outside the polder in winter and rainy seasons is another problem. Sometimes, rainwater is collected in ponds and canals for providing supplementary irrigation to the crops; however, that is also prone to salinization with the passage of the dry season [19]. In the dry season, fields remain fallow due to late rice harvest, prolonged waterlogging, high soil salinity, and unavailability of good quality water for irrigation [20, 21]. Thus, sustainable intensification of cropping systems is difficult, *albeit* an imperative in this area. We expected that the popular rice-based cropping systems must have thrived in this ecologically challenged region because of their one or more positive system outcomes which need to be compared across cropping systems.

We aimed to account for the system yield, economics, energy budgets and GHGs emission from different existing rice-based systems in the studied locations. Besides, to address the uncertainty of future GHGs emissions from the area due to climate change, we performed Monte-Carlo Simulation (MCS) with the collected data from the sampled area. Although several works have been done on the improvement of productivity and profitability of different systems, the present work is perhaps the first initiative to estimate the energy and GHGs emissions from the coastal area. Summarily, the objective of the study was to: a) estimate yield, profit, and energy input-output from different rice-based systems, b) evaluate the area- and yield-scaled GHGs emissions from those systems, and c) measure the uncertainty associated with the GHGs emission in the study area.

## Methodology

### Study area

**Fig 1:**
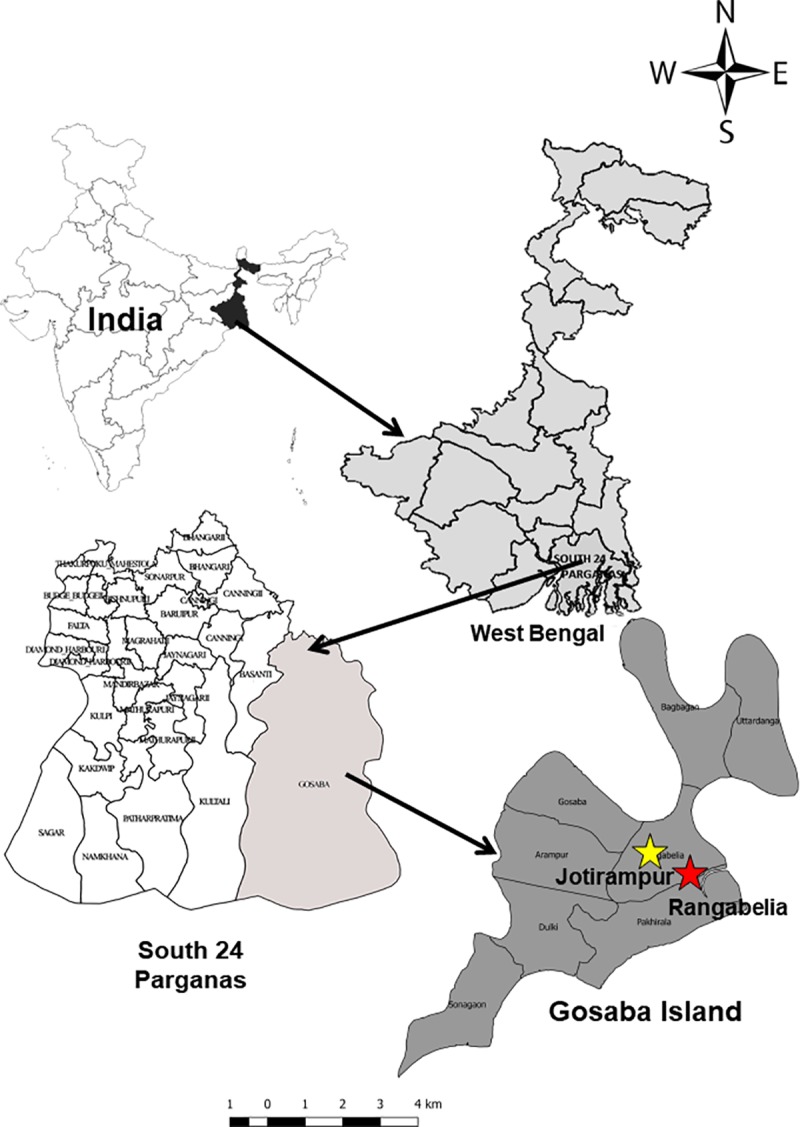
**Location of the study** [the figure is drawn from the CIA (public domain) (https://www.cia.gov/library/publications/the-world-factbook/index.html) that complies with the CC BY 4.0 license. The figure is similar but not identical to the original image and, therefore, for illustrative purposes only.].

The experiments were located at Rangabelia and Jotirampur villages under Gosaba Block, South 24 Parganas district, in the lower Gangetic plains of West Bengal (20°20' N to 20°06' S latitude and 88°20' E to 88°60' W longitude) (Fig 1). The agro-climatic condition of this area is largely influenced by the Bay of Bengal, which is nearly 35 km away from the experimental location. The climate of this coastal region is classified as hot humid with average annual rainfall varying between 1378 and 2485 mm, a major portion of which occurs between June to September [22]. The average temperature of the study area ranges between 15°C and 36°C [23], the temperature being high during the summer months and a short, mild winter during December and January. The relative humidity remains high during the period between June to October. Because of the proximity to the sea, the coastal area is exposed to nor'easter and cyclonic storms (from April to October). The cyclones at times bring in high tidal bore causing heavy loss of properties, standing crops, livestock and human lives. The farmers of the area predominantly follow rice-fallow systems; lack of irrigation water, salinity stress, soil moisture stress at planting time of winter crops, etc. are some of the main reasons for not standardizing double-crop systems in the area. Bidhan Chandra Krishi Viswavidyalaya (BCKV) and Commonwealth Scientific and Industrial Research Organization (CSIRO) of Australia have been working on intensifying rice-based cropping systems in the area since 2016 through a long-term project funded by the Australian Centre for International Agricultural Research (ACIAR). The activities are being implemented in villages where representative and diverse rice-based cropping systems and associated livelihoods were found. Based on the area of acreage, we identified 13 popular rice-based cropping systems *viz*. rice-lathyrus (*Lathyrus sativus* L.)-fallow, rice-potato (*Solanum tuberosum* L.)-fallow, rice-sunflower (*Helianthus annuus* L.), rice-fallow-fallow, rice-potato-ladies finger (*Abelmoschus esculentus* (L.) Moench), rice-lentil (*Lens culinaris* Medikus)-fallow, rice-potato-rice, rice-lentil-rice, rice-rice, rice-potato-pumpkin (*Cucurbita maxima* Duchesne), rice-pointed gourd (*Trichosanthes dioica* Roxb.), rice-potato-ridge gourd (*Luffa acutangula* (L.) Roxb.), and rice-bitter gourd (*Momordica charantia* L.). Then, 60 farm households, who followed any of these selected cropping systems for at least last five years on at least one bigha (0.13 ha) of land (Table A, in S1 File), and adopted at least one component of the above said project intervention, were included in the study [24]. Informed consent was procured from all the respondents (farmers) before starting the personal interviews. No other specific permissions were required for performing the study.The farms on which information was collected were exclusively agricultural lands witnessing farming activities for at least the last twenty years. Also, no endangered or protected species were involved/harmed during the survey work. Before conducting the study, it was rigorously reviewed and finally approved by the Institutional Ethics Committee of the Integrated Rural Development and Management Faculty Centre, Ramakrishna Mission Vivekananda Educational and Research Institute. The present study is a continuation of the work of Ray et al. [5] on the techno-economic assessment of cropping systems in different ecologically fragile areas of West Bengal state. Earlier, the study was conducted in the red and lateritic zone, which is now executed to the coastal saline zone of the state, which is fundamentally different from the socio-ecological context of the previous study.

It is also worth-mentioning that the previous work studied twelve rice-based systems in red and lateritic zone, of which only three are studied in the present work. Ten unique cropping systems are covered in the present study. To show the novelty of the present study, the search results have been documented in (Fig A, in S2 File) which demonstrates the review protocol (based on the PRISMA guidelines) for the existing literature (S3 and S4 Files) related to our work.

### Collection of data

Field data were collected from the farm households through face-to-face interviews with a semi-structured interview schedule, standardized through it's pre-testing on non-sampled respondents. The interview schedule included items on different primary (land preparation, irrigation application, sowing, harvesting, etc.) and secondary (fertilizer and pesticide application) field operations, input (seed, fertilizer, etc.) management, and economics of cultivation (cost of cultivation, gross and net returns, etc.).

### Estimation of rice-equivalent yield

The yield of different crops in the dry season was converted into rice equivalent yield (REY) [5]. Then the system yield—was calculated as per Table 1.

**Table 1:** Details of the parameters studied to estimate the productivity, profitability, energetic and GHGs emission of different rice-based cropping systems.

Parameters	Definition	Equation	Rationale of selection	Reference
Rice equivalent yield of dry season crops (REY_d_)	Yield of dry season crops are converted to equivalent rice yield based on price of the produce	${{REY}_{d}\;(t\;{ha}^{-1})=Y}_{x}.\frac{P_{x}}{P_{r}}$	To ease the explanation of the yield advantage of the component crop than its sole cropping	Ray et al. [5]
		Where, Y_x_ is the yield of a dry season crops (t ha^-1^), P_x_ is the price of the dry season crop (US $ t^-1^), and P_r_ is the price of rice (US $ t^-1^).		
System yield	Wet season rice yield plus rice-equivalent yields	$System\;yield\;(t\;{ha}^{-1}\;{yr}^{-1})=Rice\;yield\;in\;wet\;season\;(t\;{ha}^{-1})+{REY}_{d}\;(t\;{ha}^{-1})$	To compare different rice-based systems based on their yield	Ray et al. [5]
Gross return	Total economic outcome received by selling the produce	$Gross\;return\#(US\;\$\;{ha}^{-1}\;{yr}^{-1})=System^{\prime }s\;output\;(kg\;{ha}^{-1}\;{yr}^{-1})\times Output\;price\;(US\;\$\;{kg}^{-1})$	A pre-requisite to estimate the net outcome of produce	Ray et al. [17] and Ray et al. [5]
Net return	Gross benefit received minus total cost of cultivation	$Net\;return\;(US\;\$\;{ha}^{-1}\;{yr}^{-1})=Gross\;return\;(US\;\$\;{ha}^{-1}\;{yr}^{-1})-Cost\;of\;cultivation\;(US\;\$\;{ha}^{-1}\;{yr}^{-1})$	To estimate net benefit derived after incurring different costs (cost A1, A2, B1, B2 and C)	Ray et al. [17] and Ray et al. [5]
Benefit: cost ratio	Gross return divided by cost of cultivation	$Benefit\;:\;cost\;ratio=\frac{Gross\;return\;(US\;\$\;{ha}^{-1}\;{yr}^{-1})}{Cost\;of\;cultivation\;(US\;\$\;{ha}^{-1}\;{yr}^{-1})}$	To estimate the net benefit of a system per unit investment	Ray et al. [17] and Ray et al. [5]
Net energy gain	Total energy of the produce minus total energy expend	$Net\;energy\;gain\;(GJ\;{ha}^{-1}\;{yr}^{-1})=Energy\;output(GJ\;{ha}^{-1}\;{yr}^{-1})-energy\;input(GJ\;{ha}^{-1}\;{yr}^{-1})$	To account the net energy derived for each system	Ray et al. [5]
Energy ratio	Total output energy of the system divided by total input energy of the system	$Energy\;ratio=\frac{System\;energy\;output\;(GJ\;{ha}^{-1}\;{yr}^{-1})}{System\;energy\;input\;(GJ\;{ha}^{-1}\;{yr}^{-1})}$	To estimate the system energy output per unit of input energy	Ray et al. [5]
Specific energy	System energy input divided by system yield	$Specific\;energy(MJ\;{kg}^{-1})=\frac{System\;energy\;input\;(MJ\;{ha}^{-1}\;{yr}^{-1})}{System\;yield\;(kg\;{ha}^{-1}\;{yr}^{-1})}$	To assess the energy input required for producing unit system yield	Ray et al. [5]
Energy productivity	System yield divided by system energy input	$Energy\;productivity(kg\;{GJ}^{-1})=\frac{System\;yield(kg\;{ha}^{-1}\;{yr}^{-1})}{System\;energy\;input(GJ\;{ha}^{-1}\;{yr}^{-1})}$	To estimate the system yield produced per unit expanse of energy input	Ray et al. [5]
Global warming potential (GWP)	Total emission (CO_2_, N_2_O, CH_4_) from an area	$GWP\;(t{CO}_{2eq}{ha\;}^{-1}{year}^{-1})={CO}_{2}emission\times GWPof{CO}_{2}+{CH}_{4}\;emission\times GWPof{CH}_{4}+N_{2}Oemission\times GWPofN_{2}O$	To assess the impact of different systems on emission in terms of CO_2_ equivalent	Ray et al. [5]
Yield-scaled GHG emission (YSGHG)	GHG emission for per unit of system yield	$Yield\;scaled\;GHG\;emission\;of\;a\;system\;(t{CO}_{2eq}t^{-1}\;system\;yield)=\frac{GWP\;(t{CO}_{2eq}\;{ha\;}^{-1}{yr}^{-1})}{System\;yield\;(t\;{ha}^{-1}\;{yr}^{-1})}$	To estimate the comprehensive impacts of cropping practices on GHG emissions and system yields	Li et al. [31]

#1 US$ = 70.91 INR as on July 26, 2019.

### Estimation of economics

The cost of cultivation of any system (US$ha^−1^) was estimated from total expenditure to perform different field operations and input used for any system. Then the gross return, net return, and benefit:cost ratio (B:C ratio) was estimated as per Table 1.

### Estimation of energy

Input and output energy for any system was estimated by multiplying different inputs (machinery, electricity, diesel, labour, fertilizer and pesticide) and outputs (economic produce) of farm operations with their respective energy equivalents (Table B, in S1 File).Different energy indices were estimated as per Table 1.

### Calculation of GHGs emission

The Cool Farm Tool (CFT^®^), a computer software-based farm-level GHGs emission calculator [25, 26], integrates simple emission factor approaches (following Intergovernmental Panel on Climate Change or IPCC Tier 1) and process-based models requiring a higher level of data input and training (following IPCC Tier 3). It has seven different input windows *viz*. a) general information (location, the total area of the component crop of any rice-based system, the economic yield of the crop per unit area and climatic condition of the location), b) crop management (name of the crop, information about soil texture, organic matter, moisture, drainage, pH, fertilizer rate, time, dose and source, pesticide application schedule and crop residue management), c) sequestration (land use and management and above ground biomass), d) livestock (its life cycle, feed characteristics and manure management during juvenile, adult productive and non-productive phases and enteric fermentation), e) field energy use (electricity and diesel used during different field operations from different sources), f) primary processing (energy used during storage and processing from different sources), and g) transport (the type of vehicle for transporting, the distance of market from the farm and quantity of produce transported). In the results-graphs window, it calculates emission of CO_2_, N_2_O and CH_4_ in kg ha^-1^ basis from fertilizer production, direct and indirect field N_2_O, paddy methane, pesticides, crop residue management, C stock changes, livestock enteric emission, livestock manure management, livestock feed, field energy use, primary processing and wastewater use. Then it gives an estimation of global warming potential in terms of kg CO_2_ equivalent of each of the GHGs per ha basis.

The CFT^**®**^ has been highly recognised as a farm-focused spreadsheet-based GHGs calculator [25]. CGIAR has also worked on six different online GHGs emission accounting tools (CCAFS-MOT, ALU, SHAMBA, EX-ACT, CFT, and CBP), and concluded that CFT^**®**^ is freely available software that uses mixed emission models and requires moderate expertise to calculate the emission from farm level [27]. This tool has so far been successfully validated for coffee [28], wheat [29], potato production systems [30], rice-based cropping systems in West Bengal itself [5], etc. Moreover, since one of the major focuses of the present study is to judge the relative performances of different rice-based cropping systems (not the absolute measurement per se) and we have taken data at a single point of time, we may expect the climatic factors not to have a distorting effect on the relative measurements.

### Estimation of GWP and yield-scaled GHG emission

Both area-scaled (GWP) and yield-scaled GHGs emission was estimated in the present study by using the formula given in Table 1.

### Uncertainty analysis

Probable uncertainty in yield-scaled GHG emission was measured using the Monte Carlo Simulation method [32]. For this, firstly, regression analysis was performed to get a linear model to predict the contribution of inputs on the output of the study. Then through the MCS method, simulated data points of each input variable were generated to get the cumulative probability density function (CDF). The equation derived after performing the regression analysis was:

$$YSGHG=3.535-0.039\;F-0.020\;D-0.030\;S-0.166\;P+0.204\;LP-4.208\;IP\;(r=0.964;R^{2}=0.929;Durbin-Watson\;value=1.301)$$ (1)

Where, F, fertilizer; D, diesel; S, seed; P, pesticide; LP, land preparation; IP, irrigation pump.

Tornado chart was prepared to get the static sensitivity of different inputs to the output of the present study. The specifications of generating the chart in SPSS is given in the (Table C, in S1 File).

The heatmap was generated to compare the 13 cropping systems across 13 performance parameters. For this, we rescaled the value of all parameters into 0–100. Then we rendered all these values unidirectional in terms of their desirability. For example, higher emission is less desirable than lower emission and we subtracted the rescaled value of such parameters from 100.For scaling, we used the following formula–

$$RP_{i}=\frac{{(P}_{ij}-P_{il})}{\left(P_{ih}–P_{il}\right)}\times 100$$ (2)

Where, RP_i_is the rescaled value of Parameter i; *P*_*ij*_ = value of *j*th observation of *i*th parameter; *P*_*il*_ = lowest value of *i*th parameter; *P*_*ih*_ = highest value of *i*th parameter

The heatmap was generated in SPSS v.21.0 (Version 21.0, IBM SPSS Statistics for Windows, IBM Corporation, Armonk, NY, USA).

### Statistical analysis

Data on different parameters of the systems were compared by Tukey’s honest significant difference (HSD) test method. Monte-Carlo Simulation and regression analyses were used to estimate the cumulative density function of the YSGHG covering all cropping systems and to study the sensitivity of YSGHG to different inputs, respectively. All the abovesaid analyses were performed using the software SPSS v.21.0 (Version 21.0, IBM SPSS Statistics for Windows, IBM Corporation, Armonk, NY, USA).

## Results

### The productivity of rice-based systems

**Fig 2:**
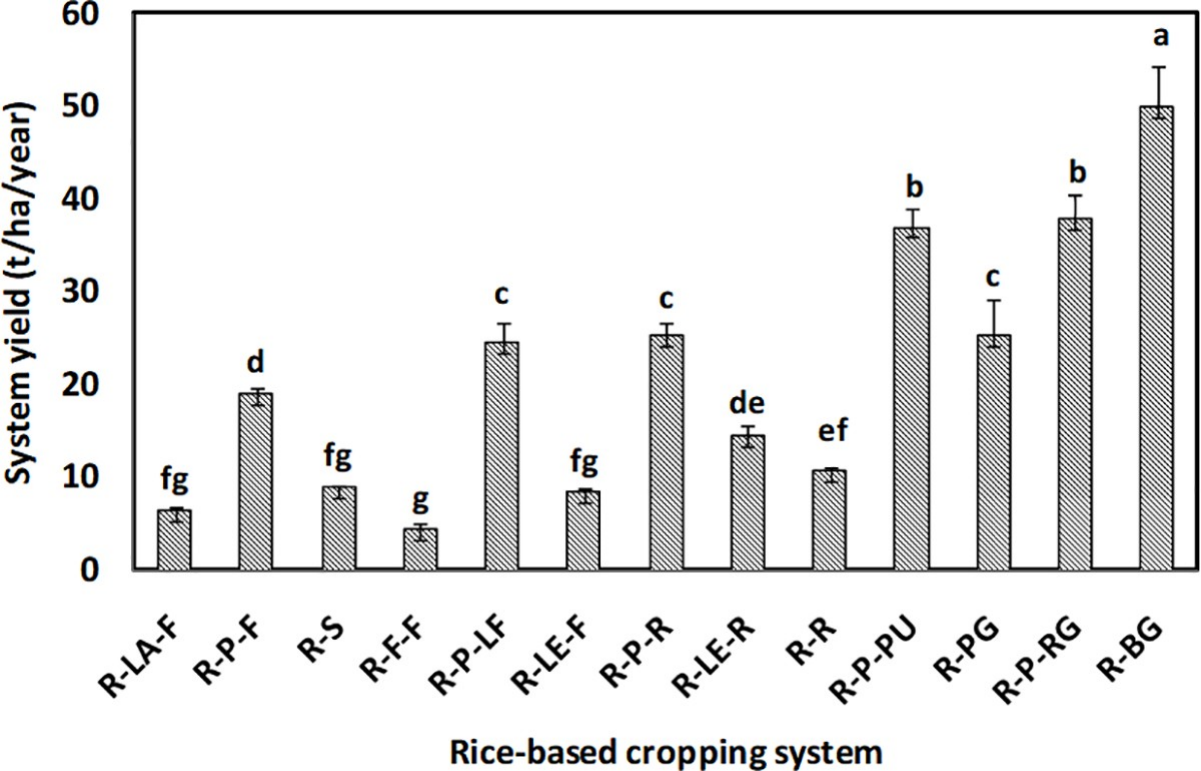
System yield (t^−1^ha) based on rice-equivalent yield of dry season crops. [R-LA-F: rice-lathyrus-fallow, R-P-F: rice-potato-fallow, R-S: rice-sunflower, R-F-F: rice-fallow-fallow, R-P-LF: rice-potato-ladies finger, R-LE-F: rice-lentil-fallow, R-P-R: rice-potato-rice, R-LE-R: rice-lentil-rice, R-R: rice-rice, R-P-PU: rice-potato-pumpkin, R-PG: rice-pointed gourd, R-P-RG: rice-potato-ridge gourd, R-BG: rice-bitter gourd]; means followed by same letters are statistically at par (otherwise significantly different at *p*<0.05) by Tukey's Honest Significant Difference (HSD) test. The standard error of means has been given as error bars. The description of the said post-hoc test also applies to Figs 3, 4, 6, 7, 8, and 10.

Significantly (*p*<0.05) highest system yield (~ 50 tha^−1^year^−1^) was estimated in the case of the rice-bitter gourd system, followed by the rice-potato-ridge gourd and the rice-potato-pumpkin systems (Fig 2). The rice-fallow-fallow system recorded the lowest system yield (< 5 tha^−1^year^−1^), followed by the rice-lathyrus-fallow and rice-sunflower systems.

### Profitability of rice-based systems

**Fig 3:**
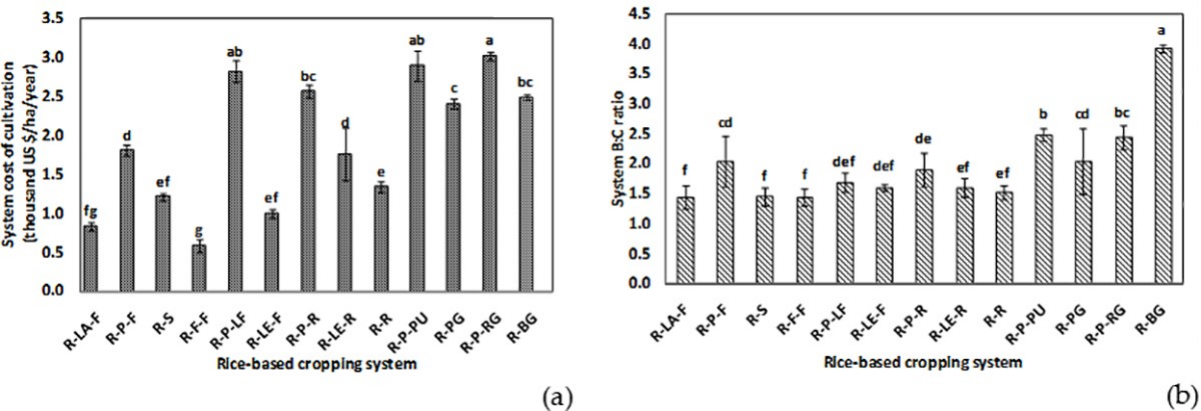
System cost of cultivation (thousand US$ha^−1^year^−1^) (a) and B:C ratio (b) of different rice-based cropping systems of the study area [R-LA-F: rice-lathyrus-fallow, R-P-F: rice-potato-fallow, R-S: rice-sunflower, R-F-F: rice-fallow-fallow, R-P-LF: rice-potato-ladies finger, R-LE-F: rice-lentil-fallow, R-P-R: rice-potato-rice, R-LE-R: rice-lentil-rice, R-R: rice-rice, R-P-PU: rice-potato-pumpkin, R-PG: rice-pointed gourd, R-P-RG: rice-potato-ridge gourd, R-BG: rice-bitter gourd].

The cost of cultivation and benefit:cost (B:C) ratio of the systems have been shown in Fig 3A and 3B. The details on gross and net return of the systems are given as a (Table D, in S1 File). Amongst the different rice-based systems, the rice-fallow-fallow system recorded the lowest cost of cultivation (~ 500 US$ha^−1^), followed by the rice-lathyrus-fallow (~ 800 US$ha^−1^) and the rice-lentil-fallow (~ 1000 US$ha^−1^) systems. Rice-potato-ridge gourd, followed by rice-potato-pumpkin (non-significant difference) and rice-potato-ladies finger systems (non-significant difference), witnessed the highest system cost of cultivation. Benefit:cost ratio was higher in rice-vegetable systems, being highest in the rice-bitter gourd (~ 4.0), followed by the rice-potato-pumpkin (~ 2.5) and the rice-potato-ridge gourd (~ 2.4) systems, respectively. Three systems *viz*. rice-fallow-fallow, rice-sunflower, and rice-lathyrus-fallow were statistically at par in recording the lowest B:C ratio (< 1.5) among all the systems studied.

### Energetics of rice-based systems

**Fig 4:**
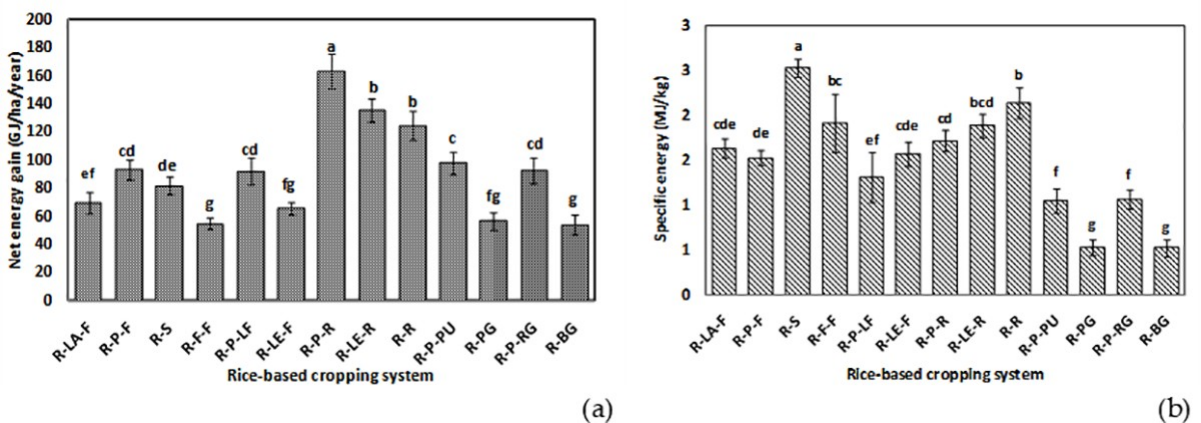
Net energy gain (GJha^−1^year^−1^) (a) and specific energy (MJkg^−1^) (b) of different rice-based cropping systems of the study area [R-LA-F: rice-lathyrus-fallow, R-P-F: rice-potato-fallow, R-S: rice-sunflower, R-F-F: rice-fallow-fallow, R-P-LF: rice-potato-ladies finger, R-LE-F: rice-lentil-fallow, R-P-R: rice-potato-rice, R-LE-R: rice-lentil-rice, R-R: rice-rice, R-P-PU: rice-potato-pumpkin, R-PG: rice-pointed gourd, R-P-RG: rice-potato-ridge gourd, R-BG: rice-bitter gourd].

**Fig 5:**
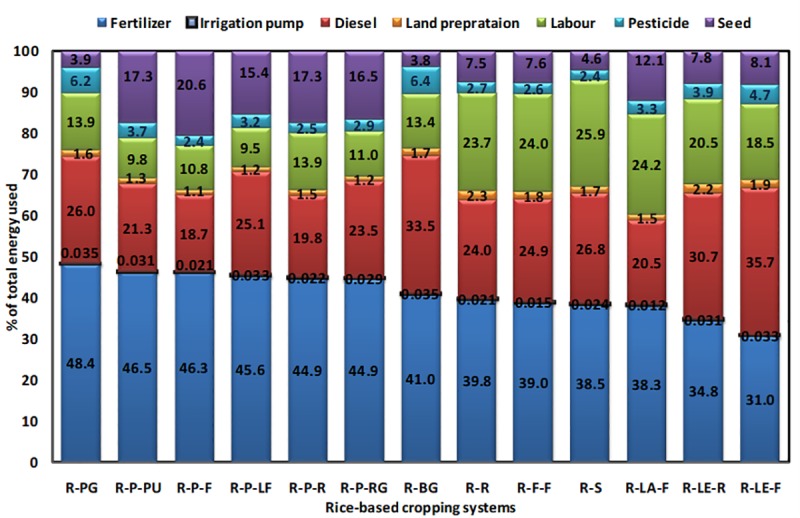
Percent energy used from different farm inputs for different rice-based cropping systems. [here, R-PG: rice-pointed gourd, R-P-PU: rice-potato-pumpkin, R-P-F: rice-potato-fallow, R-P-LF: rice-potato-ladies finger, R-P-R: rice-potato-rice, R-P-RG: rice-potato-ridge gourd, R-BG: rice-bitter gourd, R-R: rice-rice, R-F-F: rice-fallow-fallow, R-S: rice-sunflower, R-LA-F: rice-lathyrus-fallow, R-LE-R: rice-lentil-rice, R-LE-F: rice-lentil-fallow].

Fig 4A and 4B show the net energy gain and specific energy of different rice-based systems. Significantly (*p*<0.05) highest net energy gain was observed for the rice-potato-rice system (~160 GJha^−1^year^−1^), followed by the rice-lentil-rice and the rice-rice systems, respectively. In the present study, the rice-vegetable systems recorded lower specific energy. Specific energy of the rice-bitter gourd and the rice-pointed gourd systems were about 0.5 MJ^−1^kg, whereas, that of the rice-sunflower system was much higher i.e. > 2.5 MJkg^−1^, followed by the rice-rice system (> 2.0 MJkg^−1^). Data on other energy parameters are given in (Table E and Table F, in S1 File, respectively). The percent energy used by different sectors of farm operation for each rice-based system is also computed for comparing the relative contribution of different inputs/operations in different cropping systems (Fig 5). In almost all systems, fertilizer shared the major portion of the total energy used, followed by diesel and labour, respectively. The only exception was the rice-lentil-fallow system, where the share of diesel was more than that of fertilizer towards total energy use. The inclusion of potato as the second crop in the systems had increased the % contribution of seed energy to the total energy used. Across the systems, the lowest energy was shared by irrigation pump use.

### GHGs emission from rice-based systems

**Fig 6:**
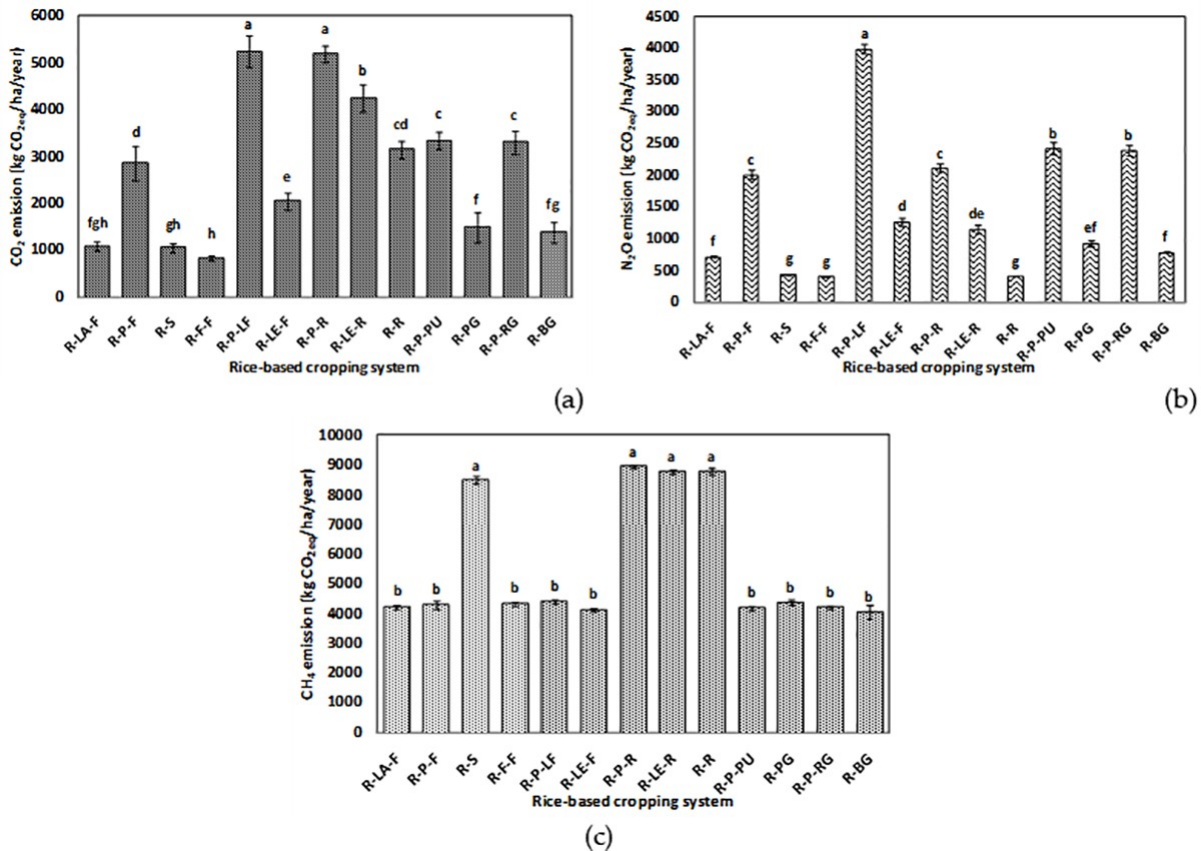
CO_2_, N_2_O and CH_4_ emission from different rice-based cropping systems of the study area. [here, R-LA-F: rice-lathyrus-fallow, R-P-F: rice-potato-fallow, R-S: rice-sunflower, R-F-F: rice-fallow-fallow, R-P-LF: rice-potato-ladies finger, R-LE-F: rice-lentil-fallow, R-P-R: rice-potato-rice, R-LE-R: rice-lentil-rice, R-R: rice-rice, R-P-PU: rice-potato-pumpkin, R-PG: rice-pointed gourd, R-P-RG: rice-potato-ridge gourd, R-BG: rice-bitter gourd].

**Fig 7:**
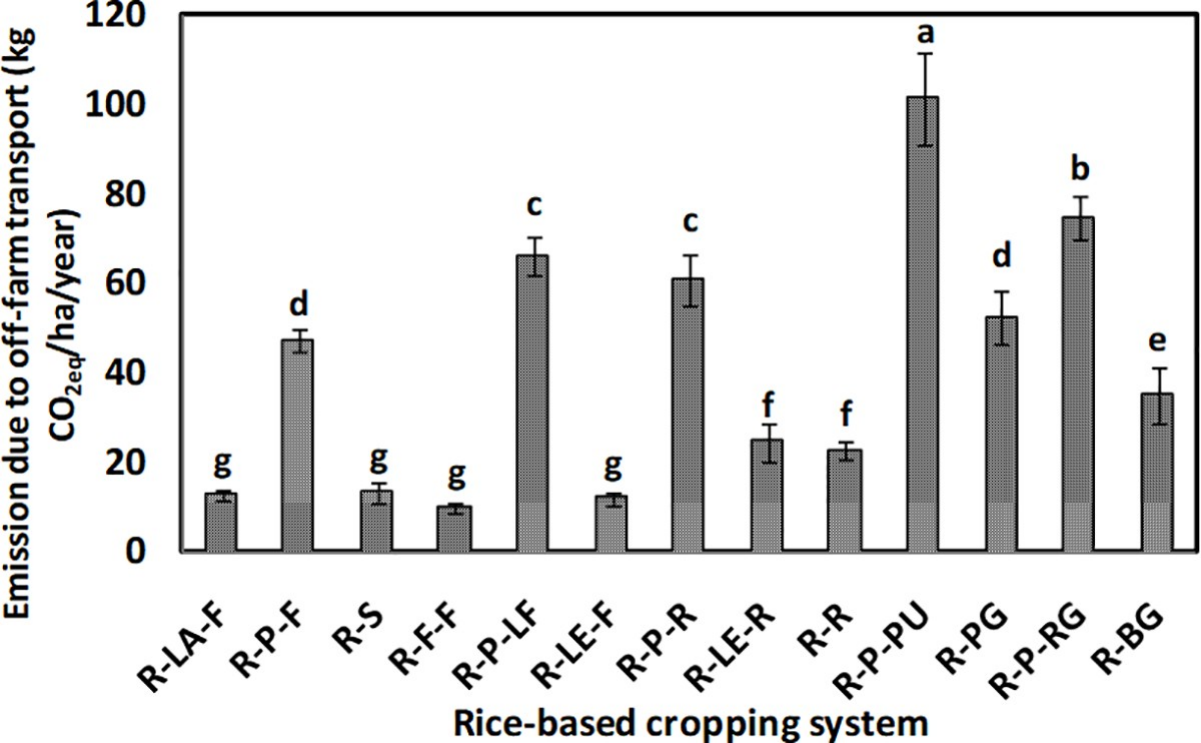
Greenhouse gases (GHGs) emission due to off-farm transport from different rice-based cropping systems of the study area. [here, R-LA-F: rice-lathyrus- fallow, R-P-F: rice-potato-fallow, R-S: rice-sunflower, R-F-F: rice-fallow-fallow, R-P-LF: rice-potato-ladies finger, R-LE-F: rice-lentil-fallow, R-P-R: rice-potato-rice, R-LE-R: rice-lentil-rice, R-R: rice-rice, R-P-PU: rice-potato-pumpkin, R-PG: rice-pointed gourd, R-P-RG: rice-potato-ridge gourd, R-BG: rice-bitter gourd.

**Fig 8:**
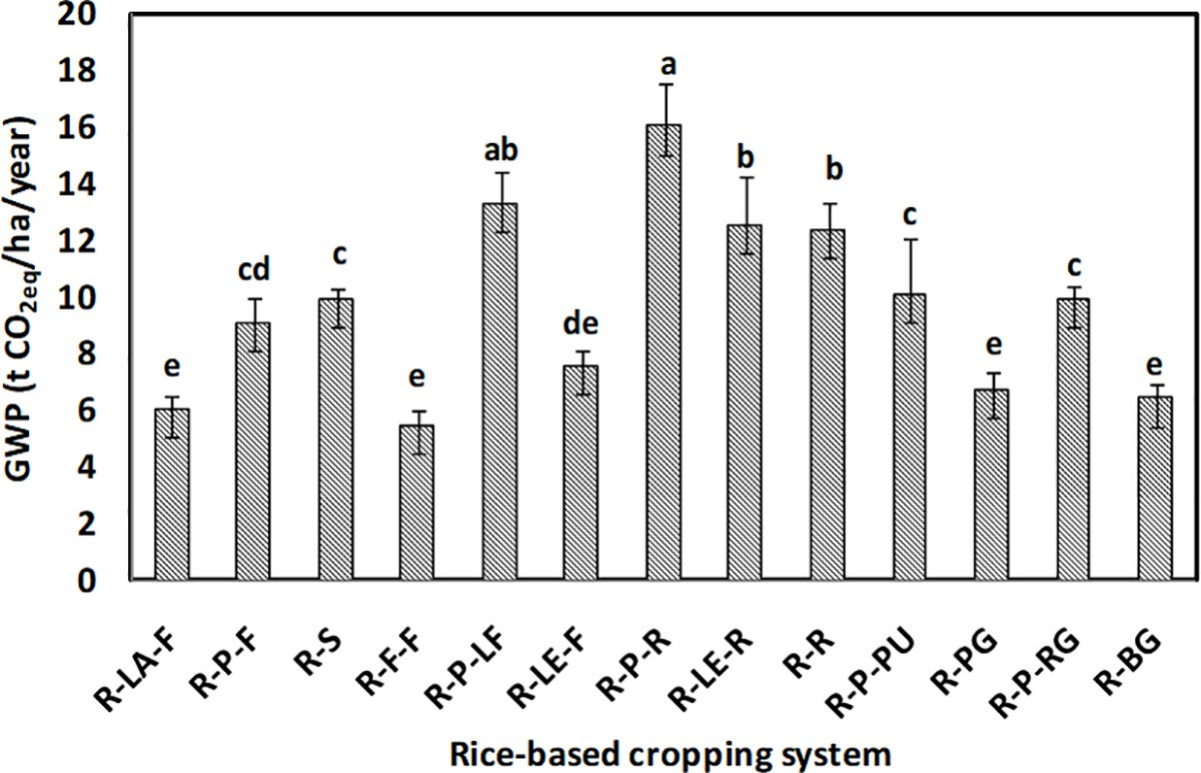
Global Warming Potential (GWP) of different rice-based cropping systems. [here, R-LA-F: rice-lathyrus- fallow, R-P-F: rice-potato-fallow, R-S: rice-sunflower, R-F-F: rice-fallow-fallow, R-P-LF: rice-potato-ladies finger, R-LE-F: rice-lentil-fallow, R-P-R: rice-potato-rice, R-LE-R: rice-lentil-rice, R-R: rice-rice, R-P-PU: rice-potato-pumpkin, R-PG: rice-pointed gourd, R-P-RG: rice-potato-ridge gourd, R-BG: rice-bitter gourd].

**Fig 9:**
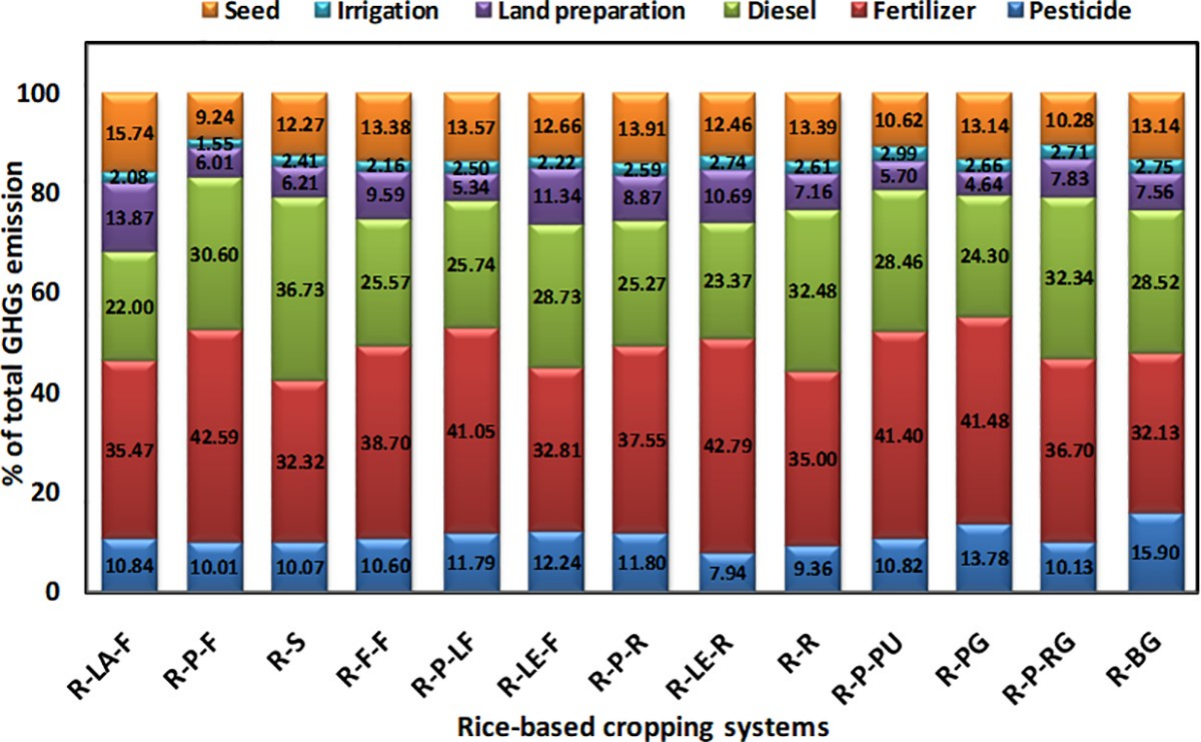
Percent emission of GHGs from different farm inputs for different rice-based cropping systems. [here, R-LA-F: rice-lathyrus- fallow, R-P-F: rice-potato-fallow, R-S: rice-sunflower, R-F-F: rice-fallow-fallow, R-P-LF: rice-potato-ladies finger, R-LE-F: rice-lentil-fallow, R-P-R: rice-potato-rice, R-LE-R: rice-lentil-rice, R-R: rice-rice, R-P-PU: rice-potato-pumpkin, R-PG: rice-pointed gourd, R-P-RG: rice-potato-ridge gourd, R-BG: rice-bitter gourd].

**Fig 10:**
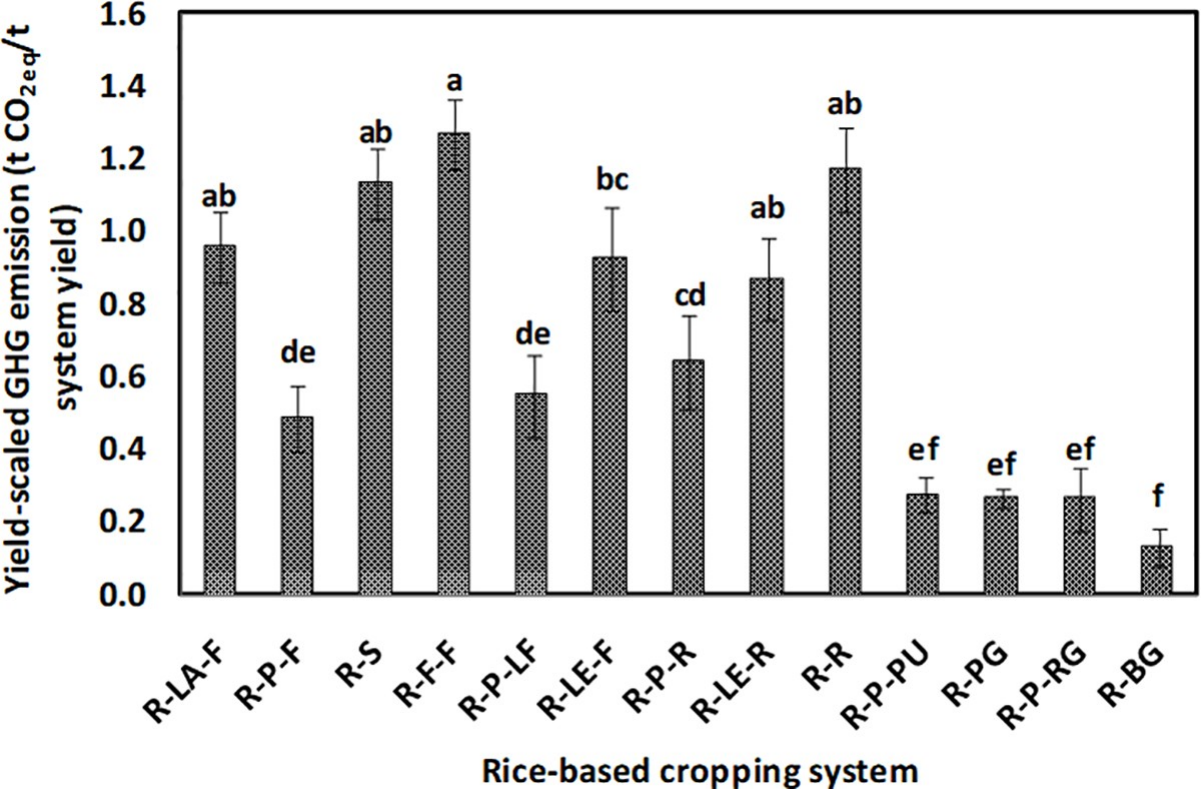
Yield-scaled GHG emission of different rice-based cropping systems. [here, R-LA-F: rice-lathyrus- fallow, R-P-F: rice-potato-fallow, R-S: rice-sunflower, R-F-F: rice-fallow-fallow, R-P-LF: rice-potato-ladies finger, R-LE-F: rice-lentil-fallow, R-P-R: rice-potato-rice, R-LE-R: rice-lentil-rice, R-R: rice-rice, R-P-PU: rice-potato-pumpkin, R-PG: rice-pointed gourd, R-P-RG: rice-potato-ridge gourd, R-BG: rice-bitter gourd].

The GHGs emission (CO_2_, N_2_O, and CH_4_) from different systems is shown in Fig 6A, 6B and 6C, respectively. The emission of CO_2_ ranged from 1,050 to 5,240 kg CO_2eq_ha^−1^year^−1^, whereas, the emission of N_2_O and CH_4_ was estimated to be about 400 to 3,950 kg and 4,050 to 8,900 kg CO_2eq_ha^−1^year^−1^, respectively. The rice-potato-ladies finger, being at par with rice-potato-rice, recorded the highest emission of CO_2_. The rice-fallow-fallow system, followed by the rice-sunflower system, experienced the lowest emission of CO_2_. An almost similar trend was observed for N_2_O emission. The rice-potato-ladies finger system was observed to have significantly (*p*<0.05) highest emission of N_2_O, followed by the rice-potato-pumpkin and the rice-potato-ridge gourd system. However, rice-sunflower, rice-fallow-fallow, and rice-rice systems were at par in terms of the lowest system N_2_O emission. Three double rice systems *viz*. rice-potato-rice, rice-lentil-rice, and rice-rice recorded the highest emission of CH_4_. These systems were statistically at par among themselves and with the rice-sunflower system in terms of their CH_4_ emission. In our study, rice-potato-pumpkin recorded significantly (*p*<0.05) the highest emission from off-farm transport (Fig 7). However, the lowest value of such emission was observed whenever there was a fallow period in the cropping systems. GWP of the systems varied between 6 to 16 t CO_2eq_ha^−1^year^−1^ (Fig 8). Significantly (*p*<0.05) highest GWP was recorded in the rice-potato-rice system, followed by the rice-potato-ladies finger, rice-lentil-rice, and rice-rice systems. Rice-vegetable systems recorded lower system GWP. The rice-bitter gourd and the rice-pointed gourd systems recorded lowest GWP, which was at par with that of the rice-fallow-fallow and the rice-lathyrus-fallow systems. System wise percent share of different emission sources to the total GWP can be envisaged from Fig 9. Irrespective of the systems, the highest emission of GHGs was from fertilizer, followed by diesel and pesticide, respectively. Yield-scaled GHG emission was estimated to curb the trade-off between the yield and GWP from the systems. It was observed that the rice-fallow-fallow system was highest in their YSGHG, followed by the rice-sunflower and the rice-rice systems (Fig 10). Amongst all the systems, rice-vegetable systems recorded lower YSGHG values, the lowest being registered in the rice-bitter gourd system.

### Risk assessment

**Fig 11:**
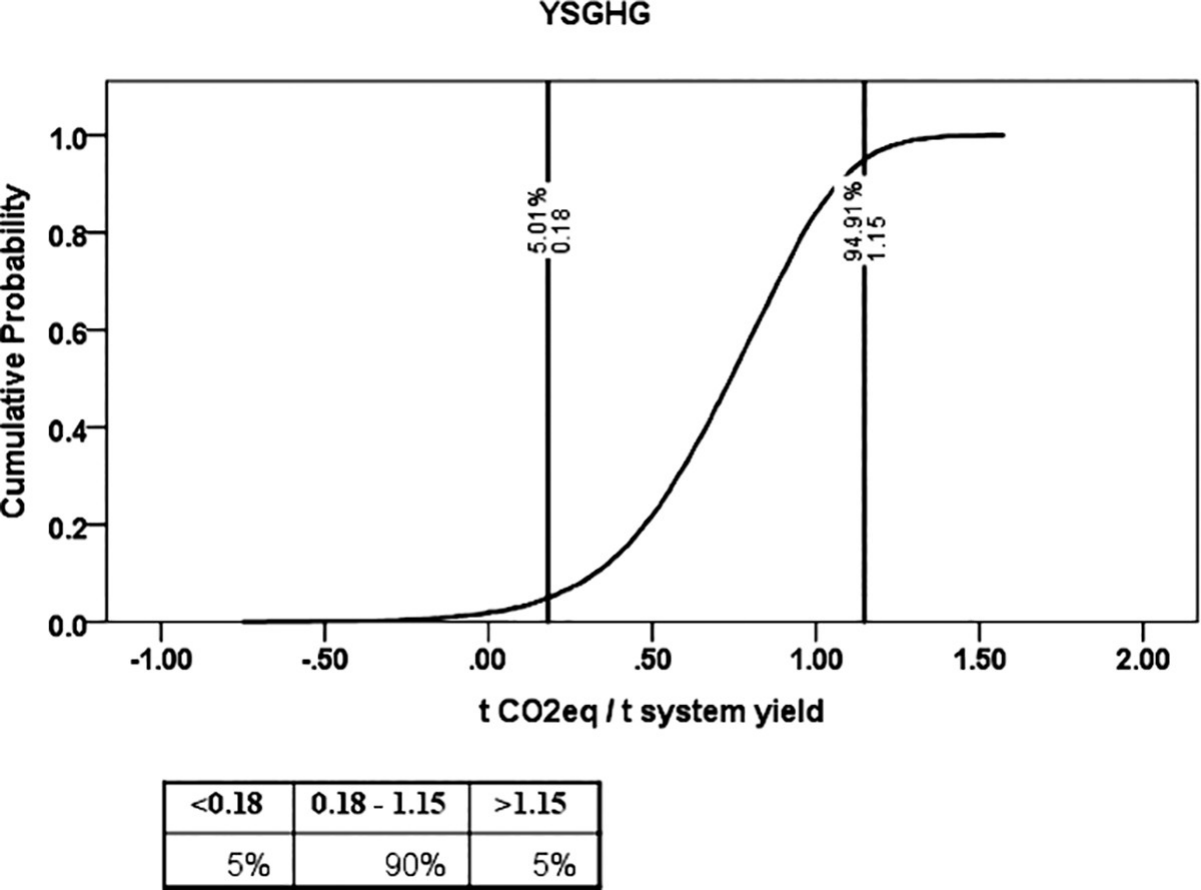
Cumulative Density Function (CDF) of yield-scaled GHG emission (YSGHG) in the study location.

**Fig 12:**
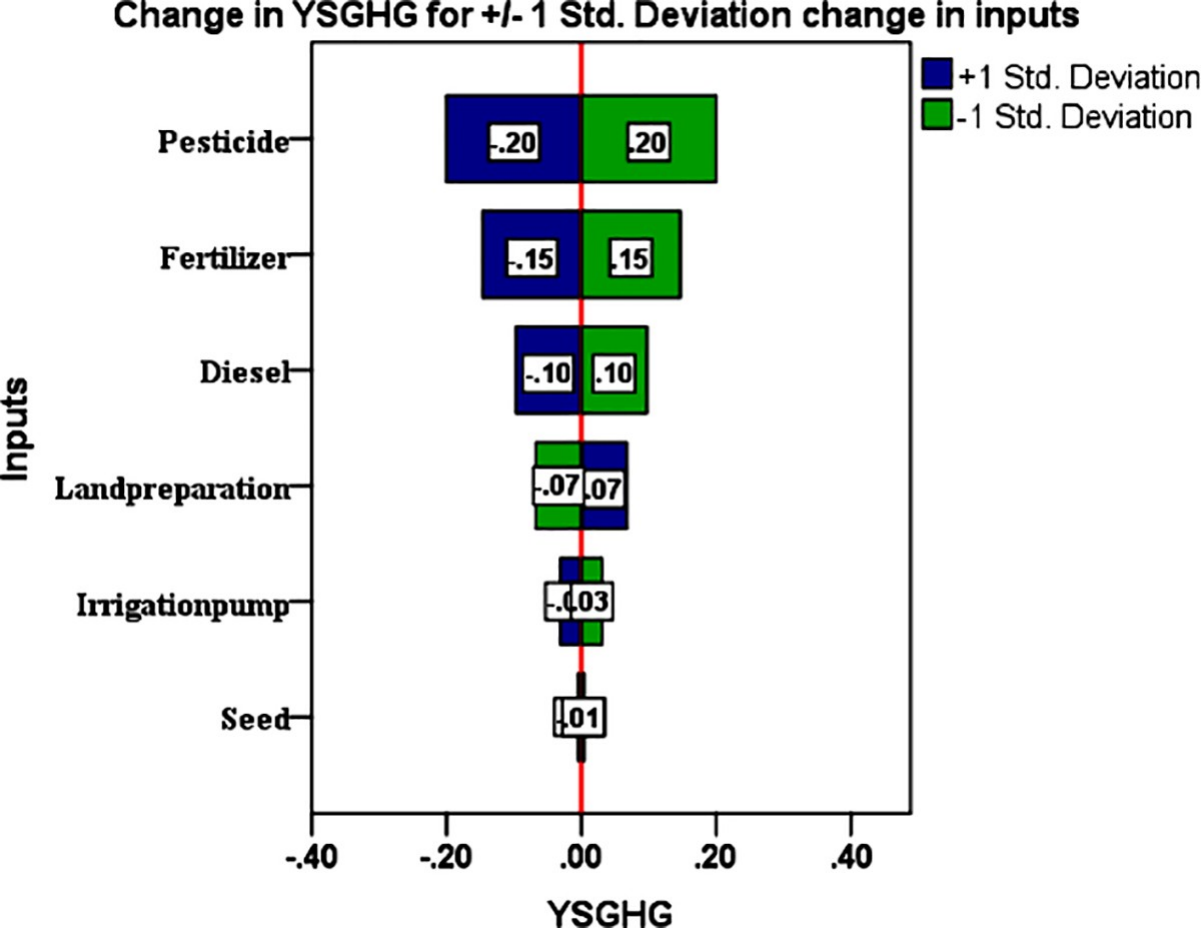
The sensitivity of different inputs to yield-scaled GHG emission (YSGHG) in the study location.

**Fig 13:**
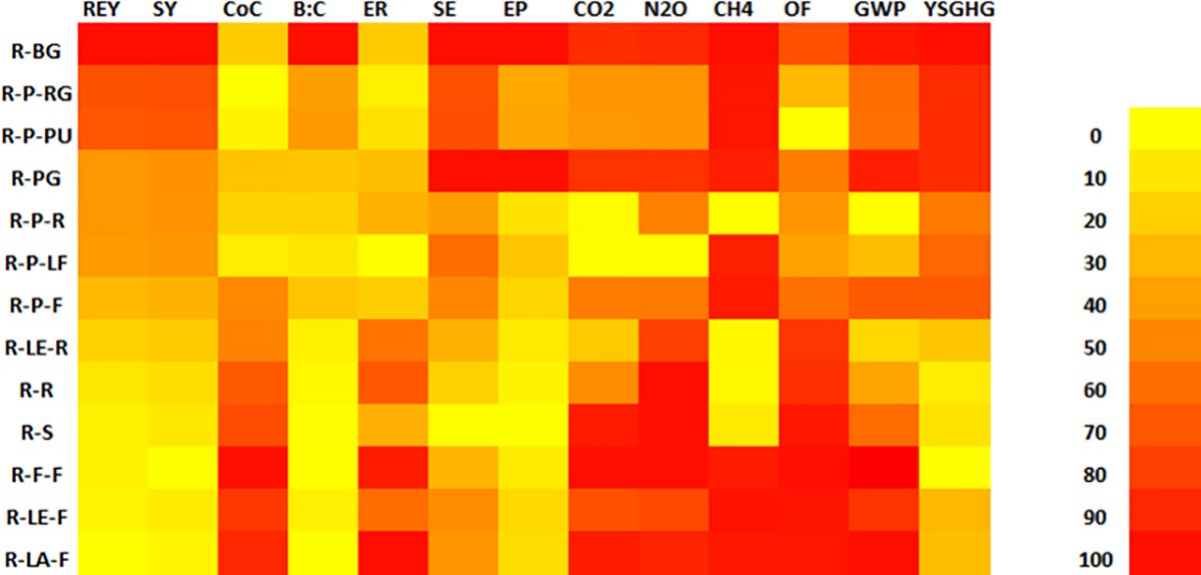
Comparison of different rice-based cropping systems based on productivity, profitability, energetics and GHGs emission indicators. Indicators are transformed into 0–100 scale for rendering comparability. [here, R-BG: rice-bitter gourd, R-P-RG: rice-potato-ridge gourd, R-P-PU: rice-potato-pumpkin, R-PG: rice-pointed gourd, R-P-R: rice-potato-rice, R-P-LF: rice-potato-ladies finger, R-P-F: rice-potato-fallow, R-LE-R: rice-lentil-rice, R-R: rice-rice, R-S: rice-sunflower, R-F-F: rice-fallow-fallow, R-LE-F: rice-lentil-fallow, R-LA-F: rice-lathyrus-fallow]; REY: rice-equivalent yield, SY: system yield, CoC: cost of cultivation, B:C: benefit:cost ratio, ER: energy ratio, SE: specific energy, EP: energy productivity, CO_2_, N_2_O, CH_4_ and OF: emission of CO_2_, N_2_O and CH_4_ and from off-farm transport, GWP: global warming potential, YSGHG: yield-scaled GHG emission.

Using Monte-Carlo Simulation, the present study estimated the cumulative density function (CDF) of YSGHG across different rice-based systems in the study area (Fig 11). It was observed that all rice-based systems taken together had a 90% probability of YSGHG emission between 0.18 to 1.15 t CO_2eq_t^−1^ system yield. There was a 5% probability of recording YSGHG emission either > 1.15 t CO_2eq_t^−1^ system yield or < 0.18 t CO_2eq_t^−1^ system yield. The sensitivity of different inputs, as observed from the tornado chart (Fig 12), shows that for +1 standard deviation increase in pesticide, fertilizer, and diesel use can cause 0.20, 0.15 and 0.10 standard deviation decreases in YSGHG. For drawing a logical conclusion about the suitability of cropping systems, a heat map of the systems based on different yield, economic, energetic and emission-related parameters is shown in Fig 13.

## Discussion

Sustainability of food production, in the context of climate change, demands the selection of suitable existing rice-based cropping systems and their improvement for a region. Some of the earlier promising rice-cereal (wheat, dry season rice, etc.) systems are now plateaued in terms of their system productivity and profitability. Few identified reasons for such plateauing are over-mining of soil nutrients, inefficient water use, pest problem, depletion of groundwater, etc. Diversification of rice-based systems in the dry season may bring some solutions to these problems [33]. Several efforts have been made to standardise site-specific suitable rice-based systems and different parameters or indicators have been used to estimate the sustainability of these systems [34]. Some of the parameters like system production and profit, energy, ecological footprint, etc. are frequently estimated on both small- and large-scales owing to their ability to judge the systems in terms of resource use efficiency. The present study has estimated some of these sustainability indicators for selected rice-based systems of the coastal saline zone with special reference to GHGs emissions.

System yield is derived from rice equivalent yield, which, is the ratio of the product of the yield and selling price of the dry season crops and the selling price of wet season rice. In the present study, the selling price of rice did not vary widely; however, the yield and price of dry season crops were different from each other. Vegetable crops had relatively higher selling price and overall yield of their economic products which might have increased their rice-equivalent yield, thereby increasing system yield of rice-vegetable systems(Fig 1). Inclusion of vegetable crops in post-rainy season rice is found to have increased system productivity [5, 35, 36]. On the other hand, we have seen that the rice-fallow system non-significantly followed by rice-lathyrus and rice-lentil systems recorded the lowest system productivity. Rice fallows, throughout South Asia, have been intensified with low water requiring pulse and oilseed crops [37]. But, the low system productivity of these systems might be due to several reasons *viz*. use of poor-quality seeds having lower yield potential in the coastal belt, delayed sowing of pulses after harvest of longer duration wet rice crops, lack of proper management by the farmers, etc. Research works by CSIRO and BCKV have already advocated suitable time for transplanting moderate-duration rice cultivars to facilitate timely sowing of pulse [24]. This would ensure a good system yield in addition to the rice-fallow intensification. That is why, the low system yield of rice-pulse / rice-sunflower systems recorded in this study, does not necessarily discourage its incorporation into existing cropping systems. Rather, it provides the scope of its improvement in the most profitable and productive way. Besides, our study also identified the rice-vegetable systems as an alternative option to be practised or fine-tuned, if necessary. These systems recorded significantly higher return (Table D, in S1 File,) and benefit:cost ratio (Fig 3) than other rice-based systems. Both in terms of productivity and profitability, rice-bitter gourd, followed by the rice-potato-ridge gourd, rice-potato-pumpkin, rice-potato-ladies finger, and rice-pointed gourd systems demonstrated best results.

The present study confirms higher net energy gain in systems having rice crops in two seasons such as rice-potato-rice, rice-lentil-rice and rice-rice systems (Fig 4). Net energy is obtained by subtracting the energy input from the energy output. It is a crucial indicator for the farmers when they have limited land to produce higher agricultural output, which is the case for most smallholder farms. Higher energy output in the rice-potato-rice systems is justified by the higher total yield of three crops and their respective energy equivalents. Such results are in good agreement with that of Ray et al. [5], who worked on different rice-based systems in the Bankura district of the West Bengal state. Higher energy use efficiency of rice-potato systems has also been confirmed by Soni et al. [38]. Higher input energy of these systems was mainly contributed by fertilizer, diesel fuel use and seed energy (Fig 5). The highest specific energy was estimated in the case of rice-sunflower system, followed by rice-rice and rice-fallow systems. This parameter is important for farmers when they need to assess the energy input required for achieving a target yield. This is important when an energy budget is to be made by a farm in advance, which is still not imminent to the farms in the study area but is essential for an accounting point of view for future researchers. This result confirms that for producing the unit amount of system yield, the highest energy was expended for these systems [5]. Lowest specific energy was estimated for the rice-vegetable systems especially for the rice-bitter gourd and the rice-pointed gourd systems. Both these systems also recorded the highest energy productivity confirming that this system produces the highest output with unit energy. Lower specific energy, on the other hand, suggested the scope of improving system efficiency by souring energy from renewable sources [39].

The order of emission of CO_2_ and N_2_O from the rice-based cropping systems was found to be–rice-potato-ladies finger = rice-potato-rice > rice-lentil-rice > rice-potato-pumpkin = rice-rice and rice-potato-ladies finger > rice-potato-pumpkin = rice-potato-ridge gourd > rice-potato-rice. On the other hand, the inclusion of a second rice (dry season) crop in the cropping systems increased the CH_4_ emission significantly. Datta et al. [10] observed higher CH_4_ emission from double rice systems due to the seedling transplanting operation and also due to the standing water situation throughout the cropping season. Tongwane et al. [40] accounted for GHGs emissions from different field crops in South Africa and observed that about 68% of the total emissions from crop cultivation were contributed by cereal crops. In our case, the highest N_2_O emission was mostly from the systems having potato crops in sequence after wet season rice crop. Higher use of fertilizer inputs in potato crops often leads to a considerable amount of N_2_O emission [41]. Higher emission of N_2_O from the rice-lentil-rice system may be due to the reduction of carbon:nitrogen (C:N) ratio of soil owing to the N-fixation by lentil crop [10]. Emissions from different rice-based systems vary due to management factors like input use, labour employment, etc., and the rice cultivars. The difference in methane emission has been found to be largely affected by the physiology of rice plants, which, in turn, is dependent on rice cultivars [42]. The present study took care of the management aspect; however, it did not typically estimate the difference in emission due to the differences in cultivars. The present study also estimated the emission from off-farm transport through CFT^®^ and was expressed in kg CO_2eq_ha^−1^year^−1^. Off-farm transport of the systems depends on several factors like the distance of the field from the market, the vehicle used for transportation, the probability of getting a good price by selling the economic products to the market [43], etc. Santos et al. [44] observed that transport emits GHGs mostly in the form of CO_2_ and road transport is, in fact, becoming a considerable factor of global warming [45]. Off-farm transportation is also included in the life-cycle assessment (LCA) due to its CO_2_ emission potential [46]. Aggestam and Buick [47] estimated off-farm transport-related emissions from the dairy farms in Sweden. Our study suggests that such vehicle emission is always associated with the higher production of fresh marketable products from crops in different systems. Inclusion of vegetables like pumpkin, pointed gourd, ridge gourd, bitter gourd, etc. increased off-farm transport from the systems. Such emissions can be managed by innovations in food distribution or promotion of the local economy like farmers’ markets [48]. The overall global warming potential was lower in such rice-vegetable systems. The same rice-vegetable systems had the lowest YSGHG values confirming the fact that these systems could be able to sustain its productivity without increasing global warming potential. Our results are in good agreement with Singh et al. [49] and Ray et al. [5]. The vice versa situation was witnessed in the rice-fallow-fallow, rice-lathyrus-fallow and rice-sunflower systems. In West Bengal, about 37.2% area under wet season rice cultivation has a subsequent fallow period in the dry season [50] Such a fallow period often blamed to have lowered the system productivity and net profit [37]. Besides, such fallow systems can have high GWP as it emits even more N_2_O than cropped field [51], perhaps due to a decrease in soil organic carbon content, thereby reducing the C:N ratio in soil [10]. However, these rice-fallow systems have great potential to be utilised further with short duration pulse or oilseed crops. Proper utilization of rice-fallows can reduce the YSGHG emission by making a good trade-off between system productivity and GWP in the study areas.

The present study also considered the uncertainty associated with the calculated yield-scaled GHGs emission from the rice-based systems. Such estimation of uncertainty is often preconditioned by the changing climatic scenario that may have some direct or indirect effect on the aberration of the GHGs emission in the future [52]. To arrive at a legible solution to this, we performed Monte-Carlo Simulation to estimate the uncertainty of GHGs emission patterns of rice-based systems. The uncertainty coupled with YSGHG emission from different rice-based systems, as is evident from CDF in Fig 11, revealed that there was a 90% probability of recording a YSGHG value of 1.15 t CO_2eq_t^−1^ system yield. Due to the absence of any normative value of YSGHG to judge its desirability for a cropping system in a region, we reported the value only as a reference for future researchers. The computed values were, however, lower than that of the estimation of Ray et al. [5] made in rice-based systems in red and lateritic soils of West Bengal. The difference in using critical inputs such as fertilizers, manures, seed, and employment of farm machinery, the preponderance of mono-cropping of rice crop (wet season), etc. may be attributed to such variation in YSGHG emission in these two regions. However, the value of YSGHG was precisely similar to the estimation of Pathak et al. [53]. In this study, there was a 5% risk of an increase in the YSGHG level above 1.15 t CO_2eq_t^−1^ system yield. Adoption of best management practices such as site-specific nutrient management, conservation tillage, sustainable intensification of rice-fallow areas, and micro-irrigation can help reduce the YSGHG level by reducing ecological footprint *vis-à-vis* sustaining the system yield [5]. Besides, there was a 5% probability of getting negative YSGHG values which signify net C sequestration from the atmosphere [54, 55]. The present farming scenario of the study area suggests that there is a strong need for fine-tuning the resource use efficiency in the existing rice-based cropping systems. Besides, the present doses of fertilizer application must be modified as per crop demands and soil test values. Dose and time of pesticide application should also be prescribed and monitored by village-level extension functionaries. Care must be taken while using diesel fuel in farm operations, be it land preparation or the application of irrigation water. The best management of these inputs may reduce GHGs emissions without compromising the yield, as was evident from the sensitivity values in Fig 12.

Consideration of a large number of parameters in 13 different cropping systems, a single-line conclusion may not be arrived at to rationalize the superiority of any system over other/s. However, they may be evaluated in retrospect by the agricultural researchers and policymakers. A heat map was developed (Fig 13) to compare the overall performances of the systems.The heat map demonstrates 13 parameters of 13 cropping systems in a single grid, where all parameters are transformed into 0–100 scale for rendering comparability.It shows that the rice-fallow-fallow and rice-lathyrus-fallow, performed better in terms of cost of cultivation, economic return, emissions (CO_2_, N_2_O, CH_4_), off-farm emission, and global warming potential, thus maintaining a balance between economy and emission. On the other hand, rice-bitter gourd performed better in terms of rice equivalent yield, system yield, B:C ratio, specific energy, energy productivity, emissions (CO_2_, N_2_O, CH_4_), global warming potential, and yield-scaled GHG, thus balancing best among productivity, energetics, and emissions.

The present study tried to estimate different parameters of system performance for different rice-based cropping systems. Although the information on system performance was recorded on existing farming practices, there are still a few limitations that may be taken care of in future researches. First, the study could be conducted in a few more villages or blocks of the coastal region for offering more precise recommendations. Second, the study could consider a few more cropping systems by involving more respondents. Hopefully, future studies in the same agro-ecological situations may address these issues, where recommendations will come out from larger sample sizes covering vast geographical regions.

Apart from the identification of optimal cropping sequences, researchers may focus on how the sub-optimal performance of cropping systems in terms of energetics and GHGs emissions can be improved by altering input managements, either by rationalising their use or by introducing a more sustainable way of sourcing them. Regarding the estimation of GHGs with CFT^®^, future researches may validate CFT^®^ measurements in relation to actual field readings e.g. by multiplying the CO_2_ emission coefficients by the application rate of inputs.

The MC analysis for all the systems can be taken up in the future, which would give insights about the emission scenarios of different rice-based cropping systems, along with the range of YSGHG. Future works can also generate more data points to reach a sound conclusion about the emission scenario in different agro-climatic zones. That will also make the classification of best, moderate and poor systems in terms of GHGs emissions and YSGHG.

## Conclusions

The present study evaluated productivity, profitability, energetics and GHGs emissions from 13 different rice-based cropping systems in the coastal saline zone of West Bengal. There was a 5% risk of YSGHG emission above 1.15 t CO_2eq_t^−1^ system yield for all 13 rice-based cropping systems in this study region.

The following conclusions may be derived from the study:

1. The rice-vegetable systems recorded better productivity, profitability, energetics and yield-scaled GHGs emission,
2. Specifically, the rice-bitter gourd system had the maximum system yield based on rice-equivalent yield and system benefit:cost ratio, while the lowest system yield and economics were recorded in the rice-fallow-fallow system.
3. Rice-sunflower, rice-rice and rice-fallow-fallow systems demonstrated the highest specific energy; the lowest specific energy was observed in the rice-bitter gourd and rice-pointed gourd systems.
4. Highest yield-scaled GHGs emission was recorded in the rice-fallow-fallow system. Rice-vegetable systems showed lower values of YSGHG.

The present study can largely dictate the desired level of productivity, profitability, and resource utilization pattern of different cropping systems of the locality. The conclusions will help the agricultural research and extension agencies, including govt. officials, NGOs, and policymakers to suggest and explain the probable best cropping systems to the farmers and what are the scopes of further improvement. They can also sensitise the farmers about the future incentives that they may receive in the form of payments on their ecosystem services.

Policymakers, in particular, have a very crucial role in the implementation of the findings of the present experiment. The identification of suitable cropping systems/cropping patterns can help them to decide the future thrust area of research, extension and agricultural planning. This will also help them identify the best management practices to be suggested to the farmers. Besides, it may also ensure the identification of the problems in implementing the best practices and its refinement through participatory approaches.

## Supporting information

S1 File (Table A) Frequencies of farmers interviewed for different cropping systems. (Table B) Equivalent energy for different inputs and outputs in the crop production system. (Table C) Script generated in SPSS for the generation of Tornado Chart. (Table D) Gross return (thousand INRha^−1^year^−1^) and net return (thousand INRha^−1^year^−1^) of different rice-based cropping systems. (Table E) Energy input (GJha^−1^year^−1^) and energy output (GJha^−1^year^−1^) of different rice-based cropping systems.(Table F) Energy ratio and energy productivity (kgGJ^−1^) of different rice-based cropping systems.
(DOCX)

S2 File (Fig A) Screening protocol used for identifying articles that report emissions from rice-based systems in coastal zones of Indian sub-continent. (The protocol followed the PRISMA guidelines. However, the review protocol was not registered or peer-reviewed. This figure is to demonstrate the existing state of literature related to the present study.).
(DOCX)

S3 File List of literature reviewed from google scholar for identifying articles that report emissions from rice-based systems in coastal zones of Indian sub-continent. (CSV)

S4 File List of literature reviewed from scopus^®^ for identifying articles that report emissions from rice-based systems in coastal zones of Indian sub-continent. (CSV)
