# Supplementary material for: Profitability, energetics and GHGs emission estimation from rice-based cropping systems in the coastal saline zone of West Bengal, India
Source: PLoS One. 2020 May 21;15(5):e0233303. doi: 10.1371/journal.pone.0233303 (PMC7241814; doi:10.1371/journal.pone.0233303)
Supplement: S1 File — (Table A) Frequencies of farmers interviewed for different cropping systems. (Table B) Equivalent energy for different inputs and outputs in the crop production system. (Table C) Script generated in SPSS for the generation of Tornado Chart. (Table D) Gross return (thousand INRha−1year−1) and net return (thousand INRha−1year−1) of different rice-based cropping systems. (Table E) Energy input (GJha−1year−1) and energy output (GJha−1year−1) of different rice-based cropping systems.(Table F) Energy ratio and energy productivity (kgGJ−1) of different rice-based cropping systems. (DOCX) [file pone.0233303.s001.docx]

**Table A.** Frequencies of farmers interviewed for different cropping systems

| **Sl. No.** | **Cropping systems** | **Frequency of farmers** |
| --- | --- | --- |
| 1. | Rice-lathyrus-fallow | 19 |
| 2. | Rice-potato-fallow | 15 |
| 3. | Rice-sunflower | 10 |
| 4. | Rice-fallow-fallow | 18 |
| 5. | Rice-potato-ladies finger | 11 |
| 6. | Rice-lentil-fallow | 11 |
| 7. | Rice-potato-rice | 9 |
| 8. | Rice-lentil-rice | 7 |
| 9. | Rice-rice | 9 |
| 10. | Rice-potato-pumpkin | 11 |
| 11. | Rice-pointed gourd | 9 |
| 12. | Rice-potato-ridge gourd | 8 |
| 13. | Rice-bitter gourd | 16 |
| Total | | 153^#^ |

#although the number of farmers interviewed was 60, the summation of frequencies of farmers practising different cropping systems is more than 60. Such result signifies that same farmer has been practising more than one of the cropping systems in their field.

**Table B.** Equivalent energy for different inputs and outputs in crop production system

| **Particulars** | **Unit** | **Equivalent energy (MJ)** | **Reference** |
| --- | --- | --- | --- |
| *Input* | | | |
| Adult man | Man-hour | 1.96 | Mittalet al. [1] |
| Adult woman | Man-hour | 1.57 | Mittal et al.[1] |
| Diesel | Litre | 56.31 | Pokhrel and Soni [2] |
| Machinery/Electric motor | kg | 64.80 | Ray et al. [3] |
| Farm machinery | kg | 62.70 | Ray et al. [3] |
| N | kg | 66.14 | Pokhrel and Soni [2] |
| P_2_O_5_ | kg | 12.44 | Pokhrel and Soni [2] |
| K_2_O | kg | 11.15 | Pokhrel and Soni [2] |
| Superior chemicals (herbicide and insecticide) | kg | 120.00 | Mittal et al.[1] |
| Micronutrient and others | kg | 10.00 | Ray et al. [3] |
| *Output* | | | |
| Rice, lathyrus, lentil | kg | 14.70 | Mittal et al. [1] |
| Sunflower | kg | 25.00 | Mittal et al. [1] |
| Potato | kg | 3.60 | Mittal et al. [1] |
| Ladies finger | kg | 1.9 | Mittal et al. [1] |
| Gourd family, pumpkin | kg | 0.8 | Mittal et al. [1] |
| Rice straw | kg | 12.5 | Mittal et al. [1] |

**Table C.** Script generated in SPSS for the generation of Tornado Chart

*Run simulation plan.

SIMRUN

/PLAN FILE='C:\Users\User\Documents\SimulationPlan_13.splan'

/CRITERIA REPRESULTS=TRUE SEED=629111597

/DISTRIBUTION DISPLAY=CDF CDFORDER=ASCENDING SCALE = PDFVIEW(CURVE) OVERLAYTARGETS(NO)

/BOXPLOT DISPLAY=NO

/SCATTERPLOT DISPLAY=YES ROWSIZE=DEFAULT

**/TORNADO DISPLAY=YES TYPE= DELTASTDDEV(1.0)**

/PRINT DESCRIPTIVES=YES(CILEVEL=95.0) PERCENTILES=NO

/OUTFILE FILE='C:\Users\User\Desktop\DATA.sav'.

**Table D.** Gross return (thousand INRha^−1^year^−1^) and net return (thousand INRha^−1^year^−1^) of different rice-based cropping systems

| **Systems** | **Gross return** | **Net return** |
| --- | --- | --- |
| Rice-lathyrus-fallow | 83.12 ± 1.766h | 25.62 ± 4.109gh |
| Rice-potato-fallow | 255.00 ± 11.779d | 130.05 ± 5.585d |
| Rice-sunflower | 121.92 ± 2.902g | 37.92 ± 4.093fg |
| Rice-fallow-fallow | 58.32 ± 2.680i | 17.82 ± 2.209h |
| Rice-potato-ladies finger | 328.60 ± 7.950c | 133.90 ± 7.598d |
| Rice-lentil-fallow | 110.82 ± 5.555g | 41.82 ± 5.652f |
| Rice-potato-rice | 337.94 ± 20.554c | 160.49 ± 8.296c |
| Rice-lentil-rice | 193.76 ± 12.701e | 72.26 ± 3.551e |
| Rice-rice | 141.26 ± 7.858f | 48.26 ± 3.309f |
| Rice-potato-pumpkin | 497.40 ± 5.469b | 297.45 ± 7.936b |
| Rice-pointed gourd | 339.57 ± 15.907c | 173.07 ± 10.285c |
| Rice-potato-ridge gourd | 510.00 ± 12.814b | 301.43 ± 15.031b |
| Rice-bitter gourd | 673.32 ± 9.877a | 501.57 ± 15.611a |

Data are means ± standard error of samples, means followed by different letter are significantly different at *p*<0.05 (otherwise statistically at par) by Tukey's Honest Significant Difference (HSD) test. 1 INR = 0.014 US$ as on July 26, 2019.

**Table E.** Energy input (GJha^−1^year^−1^) and energy output (GJha^−1^year^−1^) of different rice-based cropping systems

| **Systems** | **Energy input** | **Energy output** |
| --- | --- | --- |
| Rice-lathyrus-fallow | 10.05 ± 1.057hi | 79.53 ± 5.416f |
| Rice-potato-fallow | 28.86 ± 3.918cd | 122.13 ± 1.740d |
| Rice-sunflower | 21.41 ± 7.466efg | 103.00 ± 9.249e |
| Rice-fallow-fallow | 8.27 ± 1.693i | 63.13 ± 4.702f |
| Rice-potato-ladies finger | 40.26 ± 4.058ab | 132.32 ± 7.440cd |
| Rice-lentil-fallow | 12.89 ± 1.670hi | 78.57 ± 16.113f |
| Rice-potato-rice | 43.20 ± 2.662a | 206.22 ± 23.119a |
| Rice-lentil-rice | 27.23 ± 2.618de | 162.66 ± 10.123b |
| Rice-rice | 22.61 ± 2.753def | 147.22 ± 5.634bc |
| Rice-potato-pumpkin | 34.09 ± 2.282bc | 131.92 ± 5.681cd |
| Rice-pointed gourd | 16.02 ± 2.620gh | 72.60 ± 7.321f |
| Rice-potato-ridge gourd | 35.82 ± 5.049b | 128.50 ± 7.808cd |
| Rice-bitter gourd | 16.43 ± 1.896fgh | 70.44 ± 5.591f |

Data are means ± standard error of samples, means followed by different letter are significantly different at *p*<0.05 (otherwise statistically at par) by Tukey's Honest Significant Difference (HSD) test.

**Table F.** Energy ratio and energy productivity (kgGJ^−1^) of different rice-based cropping systems

| **Systems** | **Energy ratio** | **Energy productivity** |
| --- | --- | --- |
| Rice-lathyrus-fallow | 7.91 ± 1.412a | 611.35 ± 21.831de |
| Rice-potato-fallow | 4.23 ± 0.866ef | 654.54 ± 32.273d |
| Rice-sunflower | 4.81 ± 0.609de | 394.37 ± 25.813g |
| Rice-fallow-fallow | 7.63 ± 0.559ab | 522.30 ± 18.630ef |
| Rice-potato-ladies finger | 3.29 ± 0.369f | 765.12 ± 44.523c |
| Rice-lentil-fallow | 6.09 ± 0.881c | 636.65 ± 32.683d |
| Rice-potato-rice | 5.97 ± 0.685de | 530.14 ± 31.989de |
| Rice-lentil-rice | 4.77 ± 0.462cd | 581.46 ± 22.767ef |
| Rice-rice | 6.51 ± 0.155bc | 466.54 ± 27.844fg |
| Rice-potato-pumpkin | 3.87 ± 0.317ef | 952.99 ± 60.965b |
| Rice-pointed gourd | 4.53 ± 0.144ef | 1911.81 ± 94.807a |
| Rice-potato-ridge gourd | 3.59 ± 0.324ef | 939.32 ± 82.450b |
| Rice-bitter gourd | 4.29 ± 0.604ef | 1909.58 ± 90.017a |

Data are means ± standard error of samples, means followed by different letter are significantly different at *p*<0.05 (otherwise statistically at par) by Tukey's Honest Significant Difference (HSD) test.
