# Supplementary material for: Profitability, energetics and GHGs emission estimation from rice-based cropping systems in the coastal saline zone of West Bengal, India
Source: PLoS One. 2020 May 21;15(5):e0233303. doi: 10.1371/journal.pone.0233303 (PMC7241814; doi:10.1371/journal.pone.0233303)
Supplement: S2 File — (Fig A) Screening protocol used for identifying articles that report emissions from rice-based systems in coastal zones of Indian sub-continent. (The protocol followed the PRISMA guidelines. However, the review protocol was not registered or peer-reviewed. This figure is to demonstrate the existing state of literature related to the present study.). (DOCX) [file pone.0233303.s002.docx]

Figure S1. Screening protocol used for identifying articles that report emissions from rice-based systems in coastal zones of Indian Sub-continent. The protocol followed the PRISMA guidelines. However, the review protocol was not registered or peer-reviewed. This figure is to demonstrate the existing state of literature related to our study.
